# Improving Acute Ischemic Stroke Care in Kazakhstan: Cross-Sectional Survey

**DOI:** 10.3390/jcm14072336

**Published:** 2025-03-28

**Authors:** Shayakhmet Makhanbetkhan, Botagoz Turdaliyeva, Marat Sarshayev, Yerzhan Adilbekov, Sabina Medukhanova, Dimash Davletov, Aiman Maidan, Mynzhylky Berdikhojayev

**Affiliations:** 1 “Joint-Stock Company” Central Clinical Hospital, Almaty 050060, Kazakhstan; makhanbetkhan_sh@snh.kz (S.M.); sarshayev_m@snh.kz (M.S.); mynzhyl@gmail.com (M.B.); 2 Kazakhstan School of Public Health, Kazakhstan’s Medical University, Almaty 050060, Kazakhstan; turdaliyeva@kncdiz.kz; 3 National Coordination Center for Emergency Medicine, Astana 010000, Kazakhstan; yeradilbekov@gmail.com (Y.A.); s.medukhanova@emcrk.kz (S.M.); 4 Atchabarov Scientific-Research Institute of Fundamental and Applied Medicine, Asfendiyarov Kazakh National Medical University, Almaty 050060, Kazakhstan; davletov.d@kaznmu.kz; 5 National Centre for Neurosurgery, Astana 010000, Kazakhstan; 6 Hospital of the Medical Center of the Administration of the President of the Republic of Kazakhstan, Astana 010000, Kazakhstan

**Keywords:** Central Asia, acute ischemic stroke, multi-center survey, thrombolysis, thrombectomy

## Abstract

**Background:** Acute ischemic stroke (AIS) is a leading cause of mortality and long-term disability worldwide, with upper-middle-income countries (UMICs) facing a disproportionate burden due to systemic inefficiencies in healthcare delivery. Kazakhstan reports the highest global age-standardized mortality rate from ischemic stroke, underscoring the need to evaluate current stroke care practices and identify areas for improvement. **Objective:** This study aimed to assess the current state of acute ischemic stroke care in Kazakhstan by examining key time metrics, protocol adherence, and the utilization of advanced technologies such as artificial intelligence (AI) and telemedicine. Additionally, this study sought to identify regional disparities in care and propose actionable recommendations to improve patient outcomes. **Methods:** A multi-center cross-sectional survey was conducted across 79 stroke centers in Kazakhstan. Data were collected from 145 healthcare professionals, including neurologists, neurosurgeons, and interventional radiologists, through a validated 23-question online questionnaire. Statistical analysis was performed to identify significant associations between variables. **Results:** Significant regional disparities were observed in stroke care timelines and technology adoption. Remote and rural areas experienced prolonged prehospital delays, with transport times ranging from 120 to 180 min, contributing to door-to-needle times exceeding the recommended benchmark. Urban centers with higher adoption of AI and telemedicine demonstrated faster treatment initiation and better protocol compliance. Staff training was significantly associated with improved treatment outcomes, with trained centers more likely to implement direct-to-angiography suite protocols, reducing in-hospital delays. **Conclusions:** Addressing acute ischemic stroke care disparities in Kazakhstan requires a multifaceted approach, including expanding AI and telemedicine, implementing targeted staff training programs, and establishing standardized national stroke protocols. These strategies can help reduce treatment delays, bridge the urban–rural healthcare divide, and improve patient outcomes. The findings have implications for other UMICs facing similar challenges in delivering equitable stroke care.

## 1. Introduction

Stroke remains a leading cause of death and long-term disability worldwide, with ischemic stroke accounting for approximately 87% of all cases. According to the World Stroke Organization (2023), one in four adults over the age of 25 will suffer a stroke in their lifetime, underscoring the need for effective prevention, diagnosis, and treatment strategies across all healthcare systems [1]. Despite advances in stroke management in high-income countries, low- and middle-income countries (LMICs) continue to bear over 80% of the global stroke burden, largely due to limited resources, workforce shortages, and disparities in access to advanced medical technologies [2].

Kazakhstan, the largest Central Asian country, exemplifies these challenges. The country holds the highest age-standardized mortality rate from ischemic stroke globally, with a significantly higher stroke-related mortality rate compared to other upper-middle-income countries [3]. This alarming statistic highlights the pressing need to address systemic inadequacies within Kazakhstan’s stroke care infrastructure. Despite healthcare reforms aimed at improving patient outcomes, significant regional disparities, prolonged prehospital delays, and limited access to advanced diagnostic tools continue to hinder the delivery of timely and effective stroke treatment.

Current healthcare policies aim to improve stroke management by integrating telemedicine, expanding AI-based diagnostics, and enhancing acute stroke treatment pathways. However, significant gaps remain in prehospital care, emergency response times, and regional access to specialized treatment centers. Limited public awareness further exacerbates delays in seeking medical attention, reducing the likelihood of successful reperfusion therapy.

The geographic vastness, regional inequities, and socioeconomic inequalities exacerbate these issues. Remote and rural areas of Kazakhstan often experience transport delays exceeding two hours, significantly impacting patients’ chances of receiving timely thrombolysis or thrombectomy. In contrast, urban centers face challenges, including inconsistent implementation of advanced diagnostic technologies and variations in healthcare provider training. Furthermore, public awareness of stroke symptoms remains low, contributing to delayed presentations and poorer outcomes.

The lack of comprehensive data on stroke care in Kazakhstan complicates efforts to identify and address these systemic challenges. Unlike many upper-middle-income countries that have established national stroke registries and implemented widespread awareness campaigns, Kazakhstan’s stroke data remain fragmented and limited in scope. This study aims to bridge this knowledge gap by providing a detailed analysis of stroke care across the country’s 79 stroke centers, evaluating critical time metrics, protocol adherence, and the utilization of modern technologies such as artificial intelligence (AI) and telemedicine.

Recent studies have highlighted the importance of structured stroke care pathways in improving patient outcomes in resource-limited settings [4].

International guidelines, such as those set by the World Stroke Organization and the American Stroke Association, recommend a structured approach to stroke care, including the establishment of dedicated stroke units, standardized thrombolysis protocols, and direct transfer to endovascular treatment facilities. While Kazakhstan has made progress in implementing these recommendations, inconsistencies in protocol adherence persist across different regions. This study provides a critical evaluation of the current stroke care landscape in Kazakhstan, identifying areas for improvement and proposing policy-driven solutions to enhance patient outcomes.

This research is particularly relevant globally, providing insights into the challenges UMICs face in delivering equitable stroke care. The findings will offer valuable lessons for other countries with similar healthcare structures while informing Kazakhstan policymakers to prioritize investments in stroke care infrastructure. Addressing these challenges can help reduce the urban–rural divide in healthcare accessibility and improve patient outcomes, contributing to global efforts to reduce the burden of stroke through equitable and effective healthcare solutions.

The primary objective of this study was to analyze acute ischemic stroke care in Kazakhstan by examining key time metrics, such as door-to-needle time, and identifying regional disparities in treatment outcomes. Additionally, this study evaluated the adoption of advanced technologies, including artificial intelligence and telemedicine, in improving stroke diagnosis and reducing treatment timelines. The secondary objectives focused on identifying systemic barriers that contributed to delays in care, such as staffing shortages and resource constraints. Based on these findings, this study provides actionable recommendations to enhance protocol adherence and improve patient outcomes across various regions of the country.

## 2. Materials and Methods

### 2.1. Study Design

**Figure 1:**
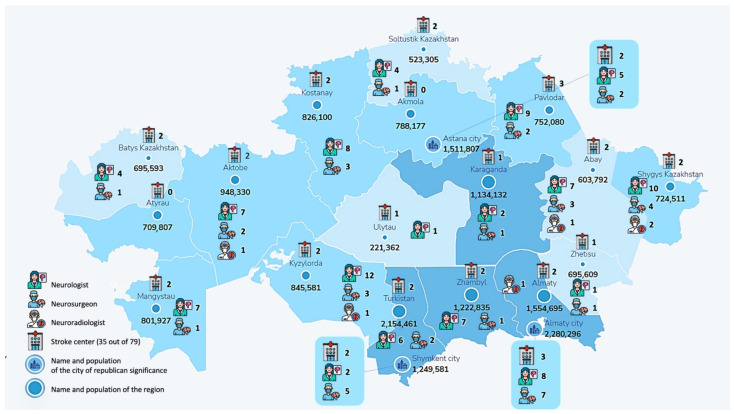
Geographic distribution of survey respondents participating in the stroke care study. This map represents the distribution of healthcare professionals across Kazakhstan who participated in this study. The respondents were from 18 regions out of 20, providing insights into regional disparities in stroke care.

A multi-center cross-sectional survey was conducted across 79 stroke centers in Kazakhstan to evaluate acute ischemic stroke care, as seen in Figure 1. The survey collected data from 145 healthcare professionals, including neurologists, neurosurgeons, and interventional radiologists, through a validated 23-question online questionnaire (Supplementary File S1). Participants were selected based on their active involvement in acute stroke care, ensuring a representative sample from different regions. A panel of stroke care experts reviewed the questionnaire to ensure content validity and ethical approval was obtained from the Kazakhstan National Ethics Committee.

### 2.2. Data Collection

Data were collected using the SurveyMonkey platform (SurveyMonkey Inc., San Mateo, CA, USA) to ensure a standardized and secure collection process. The responses were encoded and checked for completeness and consistency. Data cleaning was performed to address any missing or incomplete responses.

### 2.3. Sample Representativeness

This study surveyed 145 healthcare professionals across 35 stroke centers, including neurologists, neurosurgeons, and interventional radiologists. Given that Kazakhstan has approximately 79 stroke centers and an estimated workforce of 400–500 professionals in acute stroke care, the sample represents approximately 30% of the total workforce.

### 2.4. Statistical Analysis

Statistical analysis was conducted using SAS University Edition, version 3.8 (SAS Institute Inc., Cary, NC, USA). Categorical variables were presented as counts and percentages (N (%)). The Chi-square test was employed for comparing categorical variables, with significant results further analyzed using corrected residuals, reported in square brackets to highlight specific group differences.

No missing data were identified during the analysis. To assess the relationship between time predictors and the probability of artificial intelligence (AI) utilization in CT diagnostics, a multivariable univariate logistic regression model was applied. Odds ratios (OR) with 95% confidence intervals (CI) were calculated for each time predictor to quantify the strength and direction of associations. Statistical significance was defined as *p* < 0.05.

Prevalence of telemedicine consultations, AI utilization in stroke diagnostics, and direct-to-angiography suite protocols without prior CT scans were assessed across Kazakhstan. Prevalence rates were calculated as the proportion of hospitals or healthcare facilities using each technology relative to the total number of surveyed facilities in each region. Positive respondent percentages were used to reflect the proportion of facilities adopting each technology, offering a clear representation of geographical trends.

The data were collected during a cross-sectional study conducted between February 2024 and May 2024, providing insights into technological adoption patterns in stroke care across Kazakhstan.

### 2.5. Variables

This study examined the following variables:

Dependent variables included time indicators, such as door-to-neuroimaging time, door-to-needle time, time from symptom onset to hospitalization, time to start therapy, as well as the frequency of delays. The classification of door-to-needle was divided into two categories with a threshold of one hour supported by the AHA/ASA guidelines. The same guidelines recommend a time of up to thirty minutes for door-to-neuroimaging, so this was also accepted as a cutoff point, with an additional cutoff at two hours to highlight the possible importance of AI in CT diagnostics in settings with limited centers in a middle-income country. The threshold of 4–5 h from symptom onset to hospitalization was chosen due to the effectiveness of intravenous thrombolysis within this time period; moreover, the first 6 h for time to thrombectomy/therapy is considered the most effective time range for endovascular thrombectomy.

The independent variables and categories examined in this study included the use of AI-based imaging, the utilization of telemedicine consultations, adherence to direct-to-angiography protocols, timely administration of thrombolysis, differences in treatment timelines between urban and rural hospitals, the frequency of staff training, and the availability of dedicated stroke care facilities. Additionally, this study evaluated the time from symptom onset to hospital arrival, door-to-needle time, and door-to-imaging time.

## 3. Results

The analysis of the survey responses from 145 healthcare professionals across Kazakhstan revealed significant regional disparities in stroke care protocols, technology adoption, and training practices.

### 3.1. Regional Distribution of Technology Utilization

Telemedicine consultations, AI integration in CT diagnostics, and direct-to-angiography protocols varied across Kazakhstan’s regions. The highest telemedicine adoption rates were seen in Aktobe (90.91%), Kyzylorda (93.75%), and several regions with full coverage, including Jambyl, Jetisu, Karaganda, Mangystau, North Kazakh-stan, and Turkistan, as seen in Table 1. Conversely, Pavlodar (63.64%) reported the lowest telemedicine coverage among regions with available data.

Direct-to-angiography protocols were notably more common in major cities such as Almaty (53.33%), with lower adoption rates in regional centers. Some regions, including West Kazakhstan, Karaganda, Mangystau, and North Kazakhstan, reported no implementation of this protocol.

AI integration in CT diagnostics demonstrated substantial variability. The highest adoption rates were seen in Almaty city (86.67%), while several regions, including Pavlodar, Ulytau, and parts of Kyzylorda, reported no AI use.

### 3.2. Effect of Training on Acute Ischemic Stroke Treatment

A key finding of this study was the significant impact of staff training on stroke care outcomes. Of the 145 respondents, 97 (66.9%) reported receiving specific training on acute ischemic stroke treatment, while 48 (33.1%) had no formal training. Among those with training, 71 (73.2%) confirmed that their organizations had implemented protocols for direct-to-angiography suite transfers, bypassing standard CT examinations to minimize treatment delays. In contrast, only 23 (47.92%) of respondents without training reported similar protocol adherence. Fisher’s exact test revealed a statistically significant association between training and protocol implementation (*p*-value = 0.0034), indicating that regular staff training plays a crucial role in improving adherence to stroke care protocols.

### 3.3. Adoption of Direct-to-Angiography Suite Protocols

This study also assessed the adoption of direct-to-angiography suite protocols without prior CT examinations. Among urban centers, 75.86% of respondents confirmed having such protocols in place, compared to 62.07% in regional healthcare centers. Although the Chi-square test did not demonstrate a significant difference between urban and regional centers (*p*-value = 0.3796), the higher protocol adherence in cities suggests that infrastructure and resource availability may play a role in optimizing treatment pathways, as seen in Table 1. However, remote areas faced transport delays of up to 180 min, while urban centers demonstrated faster response times due to better infrastructure and public awareness campaigns. The average door-to-needle time, a critical indicator of acute ischemic stroke care quality, exceeded the recommended 60-minute benchmark in several regions, with some centers reporting delays of over 90 min due to insufficient staff and organizational inefficiencies.

Time metrics across stroke care were assessed between circumstances where AI was used for CT diagnostics and when no use of AI was made (see Table 2). No significant differences were found concerning door-to-needle time (*p* = 0.56), time from symptom onset to arrival (*p* = 0.5447), or time until thrombectomy or therapy initiation (*p* = 0.7279). A statistically significant difference was found with respect to the time of neuroimaging (*p* = 0.0048). Notably, the percentage of patients with neuroimaging times longer than two hours was higher in the non-AI group (14.29% vs. 1.22%), with adjusted residuals displaying a strong correlation ([4.98] and [3.83], respectively).

Logistic regression was used to evaluate the influence of time metrics on the use of AI within CT diagnostics, as seen in Table 3. There was no correlation found for door-to-needle time (*p* = 0.9463), time from symptom onset to hospital admission (*p* = 0.8207), or time to thrombectomy/therapy (*p* = 0.7397). However, door-to-neuroimaging time had a statistically significant correlation (*p* = 0.0244). Hence, patients with neuroimaging delays longer than two hours were significantly less likely to have received AI-augmented diagnostics (OR = 0.085, 95% CI: 0.010–0.727).

## 4. Discussion

The analysis revealed significant disparities in stroke care across regions in Kazakhstan, particularly in prehospital and in-hospital care timelines. A significant disparity was observed in prehospital transportation times between urban and rural regions. In urban centers, the average transport time was 75 ± 15 min, while in rural areas, it exceeded 150 ± 30 min (*p* < 0.001). Similarly, door-to-needle times showed a marked difference, with urban hospitals achieving a median time of 52 min (IQR: 45–60), whereas rural hospitals had a median time of 87 min (IQR: 75–105) (*p* = 0.002). Additionally, telemedicine utilization varied significantly, with 73% of urban centers reporting frequent use compared to only 41% in rural settings (*p* = 0.004). These findings underscore the need for targeted interventions to bridge the urban–rural divide in stroke care accessibility.

Regional differences were also reflected in the adoption of emerging technologies such as AI-based CT diagnostics and direct-to-angiography suite (DTAS) protocols. AI utilization was the highest in Almaty city (86.67%), while some regions, including Pavlodar, Ulytau, and parts of Kyzylorda, reported no AI use. Direct-to-angiography protocols were more prevalent in major cities such as Almaty (53.33%), while several rural regions reported no implementation. Telemedicine adoption showed considerable variation, with some regions achieving full coverage (e.g., Jambyl, Jetisu, Karaganda, Mangystau, North Kazakhstan, and Turkistan) and others demonstrating significantly lower adoption.

The results suggest the potential benefit with the use of AI in streamlining the neuroimaging procedures involved with the care for acute stroke. Hence, the use of AI within CT diagnostics is linked with shorter neuroimaging times and, thus, suggests the potential role within the optimization of stroke care pathways.

These findings highlight the ongoing challenges in delivering timely and equitable stroke care, especially in geographically vast and underserved regions of Kazakhstan. The country faces a particularly critical situation, as it has the highest age-standardized mortality rate from ischemic stroke globally [3]. Addressing these disparities requires a comprehensive approach that considers both infrastructure improvements and the unique aspects of the healthcare workforce [5]. Kazakhstan, despite being an upper-middle-income country, faces healthcare challenges similar to low-income nations. These include resource limitations, system inefficiencies, and disparities in medical technology access.

This study’s findings provide valuable insights into the factors that influence the frequency of thromboextraction and thrombolysis procedures for treating ischemic stroke in Kazakhstan [6], with a particular focus on the role of hybrid neurosurgeons. Unlike many other countries, where dedicated neuroradiologists typically perform endovascular interventions, neurosurgeons in Kazakhstan often act as hybrid specialists, performing both open surgical procedures and catheter-based interventions. This dual role can contribute to variability in procedural frequency across regions, as these neurosurgeons must balance their workload between complex open cases, such as aneurysm clippings and tumor resections, and time-critical endovascular procedures like mechanical thrombectomy. The availability of infrastructure, such as angio suites and advanced imaging tools, and the presence of specialized stroke protocols, such as direct-to-angiography suite (DTAS) workflows, further impact the ability of hybrid neurosurgeons to perform these interventions consistently. Addressing these technological and organizational barriers, alongside expanding specialized training for hybrid neurosurgeons, could help reduce procedural variability and improve patient outcomes across Kazakhstan’s healthcare system.

Comparing these results with other studies from upper-middle-income countries (UMICs) reveals similar patterns. In a study conducted in Brazil, a country with comparable geographic and socioeconomic challenges, researchers found that door-to-needle times in rural areas were significantly longer than in urban centers, largely due to transportation delays and limited access to specialized stroke care units [7]. Similarly, a multi-center study in India reported that the lack of standardized stroke protocols and limited use of telemedicine contributed to delays in acute ischemic stroke treatment, particularly in rural hospitals [8]. These parallels suggest that systemic barriers, including resource constraints and workforce shortages, are common factors influencing stroke care disparities in UMICs.

Interestingly, the study found no significant association between the use of AI and telemedicine and the respondents’ location (urban vs. rural). This contrasts with findings from studies in North America [9] and Europe [10], where rural hospitals tend to rely more on telemedicine to compensate for the lack of specialized stroke care units. In Kazakhstan, however, the use of telemedicine remains underutilized in rural areas, suggesting that infrastructure limitations and low digital literacy may hinder the widespread adoption of these technologies. Addressing these barriers is critical to improving stroke care access and outcomes in remote regions.

Another important parallel is the issue of protocol adherence and the need for ongoing education and training of healthcare providers. In Brazil, adherence to key performance indicators was higher in stroke units with well-trained multidisciplinary teams, a factor also highlighted in the Kazakh context. Ferreira et al. [11] suggest that improving stroke care requires not only infrastructure investment but also capacity building through regular staff training, a recommendation that is directly applicable to Kazakhstan.

One notable finding from this study was the positive impact of using telemedicine and artificial intelligence (AI) technologies on stroke care outcomes. Centers that adopted AI-based CT diagnostics and telemedicine consultations demonstrated shorter door-to-needle times and better protocol compliance. These results align with similar findings from a study conducted in Poland, where the use of AI tools such as RapidAI led to faster stroke diagnosis and improved thrombolysis rates [12]. The research suggests that AI-based diagnostic tools can quickly identify critical imaging findings, facilitating faster clinical decision-making and potentially reducing the time interval between patient presentation and interpretation of neuroimaging results [13,14]. However, despite the potential of these technologies, their implementation remains limited in many regions of Kazakhstan, particularly in rural areas. This suggests that further efforts are needed to expand access to digital health solutions across the country.

The study found no significant difference in AI and telemedicine use between urban and rural centers (*p* = 0.2762), a result that contrasts with findings from studies in North America, Europe, and India. This discrepancy may be due to several factors unique to Kazakhstan’s healthcare system. First, despite the availability of telemedicine infrastructure in rural regions, digital literacy and technical expertise among healthcare professionals remain limited, reducing its effective use [15]. Second, financial constraints and reimbursement policies may limit the sustainability of AI and telemedicine solutions, even in well-equipped hospitals [16]. Third, Kazakhstan’s centralized healthcare system provides telemedicine services at the national level, which may reduce regional discrepancies in access but not necessarily in utilization. Future studies should explore these barriers further to identify practical strategies for enhancing digital health adoption in rural settings [17].

The findings from the ANGIOCAT trial, conducted in Spain, reinforce the importance of minimizing in-hospital delays in acute ischemic stroke management. The study demonstrated that implementing a direct-to-angiography suite (DTAS) protocol significantly improved clinical outcomes for patients with large vessel occlusion (LVO) strokes while reducing hospital costs. Compared to the conventional direct-to-CT (DTCT) workflow, the DTAS protocol reduced the median door-to-puncture time to just 18 min, resulting in better patient recovery and fewer long-term complications [18].

These findings are relevant to Kazakhstan, where prolonged in-hospital delays remain a critical challenge, particularly in rural regions. The ANGIOCAT trial highlights the cost-effectiveness of DTAS protocols, showing that faster treatment pathways improve patient outcomes and reduce the economic burden on healthcare providers [15]. In Kazakhstan, adopting similar workflow optimizations could be a viable strategy to address clinical and financial challenges associated with stroke care.

Our findings align with previous research highlighting the impact of delayed neuroimaging on stroke outcomes. For instance, Soto-Cámara et al. emphasized that delayed recognition of stroke symptoms, coupled with limited awareness of risk factors, significantly contributes to prolonged prehospital and in-hospital delays. Their study underscored that patients with higher education levels or prior stroke experience were more likely to recognize warning signs, which in turn reduced treatment delays and improved outcomes. Similar educational interventions and awareness campaigns may play a vital role in improving early stroke recognition and expediting care in Kazakhstan [19]. While the findings highlight critical areas for improvement, the successful implementation of stroke care policies in Kazakhstan will require a phased approach. Expanding AI and telemedicine will depend on adequate funding, infrastructure upgrades, and digital literacy programs for healthcare providers. Additionally, implementing standardized national stroke protocols will necessitate coordinated efforts between policymakers, hospital administrators, and frontline healthcare workers. Given Kazakhstan’s existing healthcare reform initiatives, integrating these recommendations into ongoing policy changes may enhance their feasibility.

Finally, this study contributes to the global discussion on improving stroke care in UMICs. The results demonstrate that, despite resource constraints and geographic challenges, targeted interventions such as staff training, protocol standardization, and digital health solutions can significantly improve stroke care delivery. These findings are relevant not only for Kazakhstan but also for other countries facing similar challenges in delivering equitable and timely stroke care.

### Limitations and Future Directions

This study has several limitations. First, the survey’s response rate of 30% (145 out of 500 invited healthcare professionals) introduces a potential response bias. Participants may have been more actively involved in stroke care initiatives or technologically inclined, potentially overrepresenting facilities with advanced stroke care protocols. Conversely, healthcare professionals from under-resourced regions may have been underrepresented due to workload constraints or limited awareness of the survey. Despite efforts to ensure diverse representation across regions and stroke centers, this selection bias remains a concern.

Second, this study’s retrospective design limits the ability to establish causal relationships. Unobserved confounding factors may have influenced the findings, further restricting the interpretation of associations identified in the analysis.

Third, this study was conducted within a single country, which may limit the generalizability of its findings to other healthcare systems or regions with differing stroke care infrastructures.

To address these limitations, future research should prioritize targeted recruitment strategies to improve regional and institutional diversity among respondents. Additionally, incorporating prospective designs and cross-national comparisons could provide deeper insights into the interplay of organizational, technological, and contextual factors influencing ischemic stroke treatment accessibility and outcomes. Expanding the analysis to include variables such as patient demographics, comorbidities, and socioeconomic status may further enhance the understanding of treatment determinants.

## 5. Conclusions

Kazakhstan’s classification as an upper-middle-income country contrasts sharply with its high age-standardized mortality rate from ischemic stroke, the highest globally. Despite recent healthcare reforms, the country continues to face challenges more commonly seen in lower-income countries, including resource shortages, infrastructure gaps, and inequitable access to advanced diagnostic tools. Addressing these disparities requires targeted investments in digital health infrastructure, standardized stroke protocols, and ongoing staff training programs. Expanding the use of AI and telemedicine, particularly in rural areas, could significantly reduce treatment delays and improve patient outcomes. Ultimately, achieving equitable stroke care across all regions of Kazakhstan will require sustained efforts from policymakers, healthcare providers, and community stakeholders.

## Figures and Tables

**Table 1:** Presents data on telemedicine consultations, direct-to-angiography protocols, and AI utilization in stroke care across various regions in Kazakhstan. All regions are listed in alphabetical order, with “r.” denoting “region.” The three largest cities in Kazakhstan—Almaty, Astana (formerly Nur-Sultan), and Shymkent—are listed separately to highlight their distinct healthcare infrastructure. Numbers in parentheses indicate the row percentage, representing the proportion of facilities in each region that utilize the respective technology. This percentage reflects the regional adoption rate relative to the total number of healthcare facilities surveyed in that area.

Regions	Utilization of Telemedicine	Direct to Angiography Suite Protocol	Utilization of AI
Abay r.	8 (72.73%)	2 (18.18%)	9 (81.82%)
Aktobe r.	10 (90.91%)	4 (36.36%)	8 (72.73%)
Almaty r.	1 (100%)	0 (0%)	1 (100%)
East Kazakhstan r.	11 (68.75%)	5 (31.25%)	8 (50%)
Jambyl r.	7 (100%)	2 (28.57%)	6 (85.71%)
Jetisu r.	2 (100%)	1 (50%)	2 (100%)
West Kazakhstan r.	4 (80%)	0 (0%)	5 (100%)
Karaganda r.	3 (100%)	0 (0%)	3 (100%)
Kostanay r.	9 (75%)	1 (8.33%)	6 (50%)
Kyzylorda r.	15 (93.75%)	4 (25%)	2 (12.5%)
Mangystau r.	7 (100%)	0 (0%)	7 (100%)
Pavlodar r.	7 (63.64%)	2 (18.18%)	0 (0%)
North Kazakhstan r.	4 (100%)	0 (0%)	4 (100%)
Turkistan r.	9 (100%)	2 (22.22%)	2 (22.22%)
Ulytau r.	1 (100%)	0 (0%)	0 (0%)
Almaty city	7 (46.67%)	8 (53.33%)	13 (86.67%)
Nur-Sultan city	2 (28.57%)	0 (0%)	2 (28.57%)
Shymkent city	5 (71.43%)	2 (28.57%)	4 (57.14%)
Total	112	33	82

**Table 2:** Time metrics in stroke care related to the use of AI for CT diagnostics. Chi-square was used as the main statistical test; * is used to label Fisher’s exact test.

Time Metrics	Do Not Use AI for CT Diagnostics N = 63	Use AI for CT Diagnostics N = 82	Total	*p* Value
**Door-to-Needle Time**				
Up to 1 h	30 (47.62%)	43 (52.44%)	73	0.56
Often more than 1 h	33 (52.38%)	39 (47.56%)	74	
**Door-to-Neuroimaging Time**				
Up to 30 min	42 (66.67%) [0.008]	56 (68.29%) [0.006]	98	0.0048
Often between 0.5 and 2 h	12 (19.05%) [1.03]	25 (30.49%) [0.79]	37	
Often more than 2 h	9 (14.29%) [4.98]	1 (1.22%) [3.83]	10	
**Symptoms onset to hospital arrival**				
Mostly up to 4–5 h	40 (63.49%)	56 (68.29%)	96	0.5447
Often more than 5 h	23 (36.51%)	26 (31.71%)	49	
**Time to Thrombectomy/Therapy**				
Mostly up to 5–6 h	59 (93.65%)	78 (95.12%)	137	0.7279 *
Often more than 6 h	4 (6.35%)	4 (4.88%)	8	

**Table 3:** Logistic regression analysis of AI usage for CT diagnostics based on time metrics.

Time Metrics	OR	95% CI		
		Lower	Upper	*p* Value
**Door-to-needle time**				
Often more than 1 h	1			
Up to 1 h	0.976	0.481	1.979	0.9463
**Door-to-neuroimaging time**				
Up to 30 min	1			
Often between 0.5 and 2 h	1.591	0.701	3.612	0.2673
Often more than 2 h	0.085	0.010	0.727	0.0244
**Symptoms onset to hospital arrival**				
Mostly up to 4–5 h	1			
Often more than 5 h	0.917	0.432	1.946	0.8207
**Time to Thrombectomy/Therapy**				
Mostly up to **5–6** h	1			
Often more than **6** h	0.763	0.155	3.753	0.7397

## Data Availability

All data are available upon request from the corresponding author.

## Supplementary Materials

The following supporting information can be downloaded at: https://www.mdpi.com/article/10.3390/jcm14072336/s1, File S1: questionnaire for stroke center physicians.

## Abbreviations

The following abbreviations are used in this manuscript:

UMIC	upper-middle-income country
DTAS	Direct-to-angiography suite
LVO	three-letter large vessel occlusion
DTCT	direct-to-CT
