# Supplementary material for: Improving Acute Ischemic Stroke Care in Kazakhstan: Cross-Sectional Survey
Source: J Clin Med. 2025 Mar 28;14(7):2336. doi: 10.3390/jcm14072336 (PMC11990027; doi:10.3390/jcm14072336)
Supplement: Supplementary file 1 [file jcm-14-02336-s001.zip › jcm-3457254-supplementary.docx]

**QUESTIONNAIRE FOR STROKE CENTER PHYSICIANS**

**Dear Participant,**

Thank you for taking part in this survey, conducted as part of a doctoral dissertation. Your experience and opinions are an important part of our research.

The survey consists of 23 questions and will take approximately 10–15 minutes. You will be asked questions regarding practices in the treatment of acute ischemic stroke. Multiple answers may be selected for certain questions.

**Confidentiality:** All your responses will be processed anonymously and kept confidential. The survey results will be presented only in aggregated form.

**Voluntary Participation:** Participation in this survey is voluntary and free of charge. You may discontinue participation at any point without any consequences.

**Consent:** By clicking “Continue,” you confirm that you have read the information about this survey and voluntarily agree to participate.

If you have any questions or need additional information, please feel free to contact us at +7 (707) 901 1101.

Sincerely,
Shayakhmet Makhanbetkhan, Neurosurgeon, PhD Candidate, National Hospital, Almaty, Kazakhstan

1. **PLEASE INDICATE YOUR SPECIALTY:**
   - Neurosurgery
   - Neurology
   - Neuroradiology
   - Other (please specify)
2. **PLEASE INDICATE YOUR REGION:**
   - Almaty city
   - Astana city
   - Shymkent city
   - Abai Region
   - Akmola Region
   - Aktobe Region
   - Almaty Region
   - Atyrau Region
   - East Kazakhstan Region
   - Zhambyl Region
   - Zhetisu Region
   - West Kazakhstan Region
   - Karaganda Region
   - Kostanay Region
   - Kyzylorda Region
   - Mangystau Region
   - Pavlodar Region
   - North Kazakhstan Region
   - Turkistan Region
   - Ulytau Region
3. **IN YOUR ORGANIZATION, IS THERE A CLEARLY DEFINED PROTOCOL FOR ACTIONS AND COORDINATION IN PROVIDING MEDICAL CARE TO PATIENTS WITH ACUTE ISCHEMIC STROKE?**
   - No, decisions are made depending on the circumstances.
   - Yes, there is one, but it is not always possible to follow due to various circumstances.
   - Yes, in our organization, the team always works strictly according to the established protocol.
   - Unsure.
4. **DO YOU CONSULT REMOTELY (THROUGH TELEMEDICINE OR THE UNIFIED STROKE CONSULTING CENTER) OR CONSULT EXTRAMURAL SPECIALISTS IF DIFFICULTIES ARISE OR THERE IS A LACK OF SPECIALISTS IN THE DIAGNOSIS AND TREATMENT OF AIS?**
   - No, we often do not need consultation, we handle it independently.
   - Yes, but we frequently experience difficulties in finding available consultants at the required time.
   - Yes, very rarely, due to the absence of necessary conditions and convenient platforms for sharing images and clinical data.
   - Yes, we regularly use telemedicine consultations to coordinate diagnosis and treatment.
   - Other (please specify).
5. **HOW DO YOU ASSESS THE PROFESSIONALISM OF YOUR COLLEAGUES/MEDICAL STAFF IN YOUR HOSPITAL/EMS WHO PARTICIPATE IN PROVIDING MEDICAL CARE FOR ISCHEMIC STROKE?**
   - Very low—lack of necessary qualifications and training.
   - Low—they have basic knowledge but are not always able to apply it in practice.
   - Satisfactory—they possess sufficient knowledge and skills to provide care, although there can be occasional difficulties in making quick and effective decisions.
   - Good—they demonstrate a high level of professionalism, and effectively fulfill their responsibilities, but may face difficulties in complex and non-standard situations.
   - Very good—they exhibit a high level of professionalism and successfully handle most tasks, including complex situations.
6. **DO YOU PROVIDE/PARTICIPATE IN REPERFUSION THERAPY FOR ACUTE ISCHEMIC STROKE IN THE FOLLOWING WAYS?**
   - Thrombolytic therapy (TLT)
   - Thrombectomy (TE)
   - Both methods (TLT and TE)
   - Other (please specify)
7. **ON AVERAGE, HOW MANY THROMBECTOMIES/THROMBOLYTIC THERAPIES DO YOU PERFORM PER YEAR?**
   - 0–1
   - 2–5
   - 5–10
   - 10–20
   - 20 and more
8. **DO YOU USE MODERN NEUROIMAGING METHODS SUCH AS CT PERFUSION/MRI PERFUSION?**
   - Yes
   - No
   - Other (please specify)
9. **WHICH REPERFUSION THERAPY METHOD IS MORE EFFECTIVE IN YOUR PRACTICE FOR ACUTE ISCHEMIC STROKE?**
   - TLT
   - TE
   - TLT+TE (bridging therapy)
   - Unsure
10. **AVERAGE “DOOR-TO-NEEDLE” TIME**—**THE START OF THROMBOLYTIC THERAPY FROM THE MOMENT THE PATIENT ARRIVES AT THE EMERGENCY DEPARTMENT:**
    - Usually within 1 hour.
    - Usually over 1 hour.
    - Usually more than 2–3 hours.
11. **AVERAGE “DOOR-TO-IMAGING” TIME**—**THE START OF NEUROIMAGING FROM THE MOMENT THE PATIENT ARRIVES AT THE EMERGENCY DEPARTMENT:**
    - Usually within 30 minutes.
    - Usually over 30 minutes.
    - Usually more than 1 hour.
    - Usually more than 2–3 hours.
12. **AVERAGE TIME OF PATIENT ARRIVAL FOR URGENT THROMBECTOMY/THROMBOLYTIC THERAPY FROM THE ONSET OF SYMPTOMS:**
    - Usually within 1 hour.
    - Usually within 2–3 hours.
    - Usually within 4–5 hours.
    - Usually within 5–6 hours.
    - Usually after 5–6 hours.
13. **AVERAGE TIME TO INITIATE THROMBECTOMY/THROMBOLYTIC THERAPY AFTER ARRIVAL AT THE EMERGENCY DEPARTMENT:**
    - Usually within 1–2 hours.
    - Usually within 3–4 hours.
    - Usually within 5–6 hours.
    - Usually after 5–6 hours.
14. **IS THERE A PROTOCOL FOR THE DIRECT TRANSPORT OF PATIENTS WITH ACUTE ISCHEMIC STROKE FROM THE EMERGENCY DEPARTMENT TO THE ANGIO-SUITE WITHOUT PRIOR CT EXAM?**
    - No, we do not practice this and are not familiar with it.
    - Yes, but we do not have an angiographic machine (with expert CT).
    - Yes, but it is not applied in practice.
    - We practice it occasionally.
    - It is used systematically.
15. **MAIN REASONS FOR DELAYS IN THROMBECTOMY/THROMBOLYTIC THERAPY:**
    - Delay in patient arrival at the hospital (late admission, outside the therapeutic window).
    - Delay at the hospital (physician examinations, lab tests, CT/MRI diagnostic procedures).
    - Lack of specialists or absence of a team (radiologists, neurologists, neurosurgeons on assignment, on leave, etc.).
    - Insufficient instruments, supplies, or medications.
    - Other (please specify).
16. **DO YOU USE MODERN ARTIFICIAL INTELLIGENCE TECHNOLOGIES FOR STROKE DIAGNOSIS BASED ON CT SCANS SUCH AS RAPID, OLEA ISCHEMIA VIEW, RADIOMIX AND MACHINES, CEREBRA, ETC.?**
    - No, this is the first time I’m hearing about it.
    - No, it has not been implemented at our clinic yet.
    - Yes, but not often (used as an auxiliary tool).
    - Yes, it significantly influences diagnosis and treatment.
17. **WHICH OF THE FOLLOWING AI PLATFORMS HAVE YOU USED IN YOUR PRACTICE?**
    - RapidAI
    - Olea Ischemia View
    - RadiomiX and machines
    - Cerebra
    - Have not used any of the listed AI platforms
    - Other (please specify)
18. **IN YOUR CLINICAL PRACTICE, HOW EFFECTIVE ARE MODERN AI TECHNOLOGIES (RAPID, OLEA ISCHEMIA VIEW, RADIOMIX AND MACHINES, CEREBRA, ETC.) IN TREATING ACUTE ISCHEMIC STROKE? (SELECT ALL THAT APPLY)**
    - No practical benefit.
    - For independent CT image visualization via their application.
    - Accelerates diagnostic procedures.
    - Provides scale assessment and determines the volume of the lesion.
    - Enables online consultations by sharing CT images.
19. **HOW DO YOU ASSESS THE ACCURACY AND EFFECTIVENESS OF AI SYSTEMS LIKE RAPID, OLEA ISCHEMIA VIEW, RADIOMIX AND MACHINES, AND CEREBRA IN INTERPRETING CT IMAGES?**
    - Insufficient: The system regularly makes errors that can mislead clinicians.
    - Satisfactory: From time to time, the system provides erroneous results and requires constant additional verification.
    - Good: The system reliably identifies key pathological changes with minimal instances of overdiagnosis.
    - High: The system consistently identifies even the most complex pathological changes.
20. **BASED ON YOUR EXPERIENCE, WOULD YOU RECOMMEND THE IMPLEMENTATION OF AI (RAPID, OLEA ISCHEMIA VIEW, RADIOMIX AND MACHINES, CEREBRA) IN STROKE CENTERS?**
    - No, we can manage without AI.
    - Yes, AI can be useful, but there are other higher-priority areas for improvement.
    - Yes, AI could significantly enhance some aspects of our work, especially in rapid diagnosis and decision-making.
    - Strongly recommend, the potential is huge. AI is critically important for improving the efficiency and quality of treatment in stroke centers.
21. **WHAT ARE THE MAIN DIFFICULTIES IN COORDINATION BETWEEN DIFFERENT DEPARTMENTS, STROKE CENTERS, AND EMS IN PROVIDING CARE TO PATIENTS WITH ACUTE ISCHEMIC STROKE?**
    - Lack of interaction between departments/hospitals.
    - Delayed transfer of information/documentation/paperwork.
    - Absence of clear interaction protocols.
    - Difficulties in determining further strategies/types of reperfusion therapy.
    - Other (please specify).
22. **DOES THE LEVEL OF PERSONNEL TRAINING AFFECT THE SPEED AND QUALITY OF REPERFUSION THERAPY?**
    - Yes, it significantly affects it.
    - Yes, but not always.
    - It does not have a significant impact.
    - Unsure.
23. **HOW OFTEN IS TRAINING AND EDUCATION ON CURRENT METHODS OF TREATING ISCHEMIC STROKE CONDUCTED AT YOUR CENTER?**
    - Regularly (at least once a quarter).
    - Periodically (once or twice a year).
    - Rarely (less than once a year).
    - Not conducted.
